# 503. In vitro Evaluation of Sitagliptin-HIV-1 Trans-activator Transcription Peptide Nano-formula for Antiviral Activity Against SARS-CoV-2: Drug Repurposing Approach

**DOI:** 10.1093/ofid/ofab466.702

**Published:** 2021-12-04

**Authors:** Khalid Eljaaly, Hani Asfour, Tarek Ibrahim, Osama Ahmed, Nabil Alhakamy, Usama Fahmy, Mohammed Al-Rabia, Ahmed Aloafi, Mohamed Tantawy, Khulood Hussein, Ahmed Aldarmani, Mahmoud Elfaky

**Affiliations:** King Abdulaziz University, Jeddah, Makkah, Saudi Arabia

## Abstract

**Background:**

The outbreak of COVID-19 pandemic in China regarded as a major health/economic hazard. The importance of coming up with mechanisms for preventing or treating COVID-19 has been felt across the world. This work aimed at examining the efficiency of Sitagliptin (SIT) and human immunodeficiency virus type 1 (HIV-1) trans-activator transcription peptide (TAT) against SARS-CoV-2.

**Methods:**

SIT-TAT nano-conjugates were prepared according to a full three-factor bi-level (2^3^) factorial design. SIT concentration (mM, X1), TAT concentration (mM, X2), and pH (X3) were selected as the factors. Particle size (nm, Y1) and zeta potential (mV, Y2) were assessed as responses. Characterization of the optimized formula for Fourier-transformed infrared (FTIR) and Transmission electron microscope was carried out. In addition, IC50 in Vero E6 cells, In vitro 3CL-protease inhibition and docking tests were investigated.

**Results:**

The prepared complex’s formula was as follows 1: 1 SIT: TAT molar ratio, whereas zeta potential and particle size values were at 34.17 mV and 97.19 nm, respectively. This combination did exhibit its antiviral potentiality against SARS-CoV-2 via IC50 values of 9.083 5.415, and 16.14 µM for TAT, SIT-TAT, and SIT, respectively. In addition, the complex SIT-TAT showed a significant (P < 0.001) viral-3CL-protease inhibitory effect (IC50 = 3.959 µM ± 0.011) in comparison to isolated components (IC50 = 10.93 µM ± 0.25) and TAT (IC50 = 8.128 µM ± 0.42). This was further confirmed via in silico study. Molecular docking investigation has shown promising binding affinity of the formula components towards SARS-CoV-2 main protease (3-CL).

**Conclusion:**

While offering significant binding interactions with protein’s key pocket residues, an optimized formulation of SIT-TAT could guarantee both the enhanced delivery to the target cells and the improved cellular uptake. The presented findings would guarantee further investigations regarding formula optimization against SARS-CoV-2.

**Disclosures:**

**All Authors**: No reported disclosures

